# Time Trends of Greenspaces, Air Pollution, and Asthma Prevalence among Children and Adolescents in India

**DOI:** 10.3390/ijerph192215273

**Published:** 2022-11-18

**Authors:** Sowmya Malamardi, Katrina A. Lambert, Attahalli Shivanarayanaprasad Praveena, Mahesh Padukudru Anand, Bircan Erbas

**Affiliations:** 1 Department of Public Health, School of Psychology and Public Health, College of Science Health and Engineering, La Trobe University, Melbourne, VIC 3086, Australia; 2 Department of Respiratory Medicine, JSS Medical College, JSS Academy of Higher Education & Research (JSSAHER), Mysore 570015, India; 3 Department of Studies in Statistics, University of Mysore, Mysuru 570006, India; 4 Faculty of Public Health, Universitas Airlangga, Surabaya 60115, Indonesia

**Keywords:** asthma, greenspace, air pollutants, particulate matter

## Abstract

The prevalence of childhood asthma contributes to the global burden of the disease substantially. Air pollution in India has increased. In this study, we examine the associations among greenspaces, air pollution, and asthma prevalence in children and adolescents over a large, diverse population in India. We used state-wide global burden of disease data on asthma from age 0 to 19 years in 2005, 2011, and 2017. For greenspace, we used the normalized differential vegetation index (NDVI), which is the surface reflectance of light during photosynthetic activity. NDVI, air pollutants (PM_2.5_, PM_10_, SO_2_, NO_2_, and O_3_), weather, and socio-demographic factors were included in generalized estimating equation (GEE) models to estimate their associations with childhood asthma prevalence over time. Novel data visualization illustrated the complex spatial distributions. NDVI was associated with asthma prevalence (β = 0.144; 95% CI = 0.10, 0.186; *p* < 0.0001) for high PM_2.5_, along with high levels of both gaseous air pollutants, SO_2_, and NO_2_ ((β = 0.12; 95% CI = 0.08, 0.16; *p* < 0.0001) and (β = 0.09; 95% CI = 0.05, 0.13; *p* < 0.0001)). However, NDVI and high O_3_, had a strong negative association with asthma prevalence (β = −0.19; 95% CI = −0.26, −0.11; *p* < 0.0001). We observed additional effects of the interaction between the NDVI and high concentrations of PM_2.5_, PM_10_, NO_2_, and O_3_, assuming that these associations share a common pathway, and found interaction effects for asthma prevalence. Given the changing environmental conditions that interplay over geographical characteristics on the prevalence of asthma, further studies may elucidate a better understanding of these complex associations.

## 1. Introduction

Asthma is an important public health respiratory disease affecting 1–18% of the population in various countries [1]. Asthma ranks as the second most prevalent disease and the second leading cause of mortality among chronic respiratory diseases [2]. In children, the global prevalence of asthma varies from 5 to 20%, contributing substantially to the disease burden [3,4]. If uncontrolled, it results in persistent symptoms associated with asthma, increased hospitalization, reduced lung functions, and impaired quality of life [5,6].

Asthma is multifactorial, and we are yet to understand the causal mechanisms fully; however, genes, the surrounding environment, and behaviors play a part in asthma development [7,8,9]. Environmental factors may account for the substantive variations in asthma prevalence across different regions of the world [9,10,11,12]. Air pollutants, especially from traffic emissions (TRAP), lead to the worsening of existing respiratory symptoms [13,14,15] and increase the risk for new-onset asthma [16]. Globally, 5–10 million and 9–23 million annual asthma emergency room visits (ERVs) were attributable to particulate matter of diameter 2.5 µm or less (PM_2.5_) and ozone (O_3_), respectively [17,18]. Each year, 16 new million pediatric asthma cases occur due to PM_2.5_, and about 4.5 million premature deaths were attributable to air pollution itself [19,20]. Ambient air pollution due to PM_2.5_ contributes to 50% of the global total disease burden greatly in major developing countries (China and India) and 17.4 million DALYs in children younger than five years globally [21]. 

Most of the observational studies on ambient air pollution (AAP) and asthma focus on Australia, Europe, and the US [22,23,24] but very little in the South Asia region, especially in India. The estimated mortality rates from ambient air pollution (AAP) and asthma are the greatest in the South Asian subcontinent, and India alone accounted for more than 1.09 million deaths due to AAP in 2015 [21,25]. The average concentration in India of PM_2.5_ [65.2 (78.61–96.56) µg/m^3^] is 6 times more than the WHO permissible limits for PM_2.5_ (10 µg/m^3^) [26].

There is considerable interest in understanding the effect of the natural green environment on asthma, but the evidence is unclear [14,27,28]. A study in Spain observed a 60% higher prevalence of asthma in children living close to greenspaces such as parks [28], while another study from New York observed that an increased level of greenness was associated with a 17% higher prevalence of asthma in children aged 4–7 years [29], and another showed declines in prevalence among pre-school children in a New York study [27]. A recent review of studies found an association between urban greenspaces and asthma among children, although findings were inconsistent, and there was no conclusive interpretation [30]. When both greenspace and air pollution effects were studied together, the impacts of air pollution on asthma prevalence were lesser for children living in areas with higher levels of greenspaces and increased access to greenspaces [27], while this was contradicted in other studies showing an increase in asthma prevalence [31,32].

Studies in the US, Canada, and Europe have assessed both greenspace and air pollution with childhood asthma prevalence [27,28,29,32,33], but no studies have discussed South Asia. There are almost half a billion children in India, and about 6% of the children in India have asthma [10]. India comprises approximately 18% of the global population [34], and it is quite heterogeneous; diversity exists across states in environmental and topographical features. There are no studies on greenness and asthma prevalence in children and adolescents across all states and union territories in India. Studies on air pollution globally estimated the exposure rate is highest in India [21,35], and 14 of the top 20 world’s most polluted cities in terms of particulate matter of PM_2.5_ are in India [36,37]. India enormously contributes to global asthma emergency room visits due to air pollutants PM_2.5_ (30%), O_3_ (23%), and nitrogen dioxide (NO_2_) (15%) [17]. Over the last two decades, air pollution has increased in India, and particulate matter contribution to AAP is estimated to increase in India over the next ten years drastically [38,39]. In this study, we sought to estimate the level of greenness in each state of India and its association with asthma prevalence in children and adolescents. We also assessed the role of air pollutants (PM_2.5_, PM_10_, SO_2_, NO_2_, and O_3_) in these associations. We hypothesized that exposure to increased greenness over time shows lower asthma prevalence in children and adolescents. Increased levels of air pollutants in these areas will modify the association.

## 2. Materials and Methods

### 2.1. Study Design and Population

India is a diverse country spread across 15,200 km of land and 7517.24 km of coastline, and it is home to 1.3 billion residents. India comprises 29 states and 7 union territories. The health system in India operates at the state level [40], and so we present the results of an analysis of the 29 states and 1 union territory—Delhi, the national capital. The remaining six small union territories (Andaman and Nicobar Islands, Chandigarh, Dadra and Nagar Haveli, Daman and Diu, Lakshadweep, and Puducherry) were not included due to the non-availability of data for all the study variables.

### 2.2. Data Collection and Measurements

#### 2.2.1. Outcome: Asthma Prevalence Data

The prevalence of asthma data by location, age, and year we derived from the Global Burden of Disease (GBD) data visualization tool developed by the Institute for Health Metrics and Evaluation (IHME), USA [41]. The state-wise prevalence of asthma was estimated using the terms defined in the GBD studies [42,43,44]. We calculated asthma prevalence estimates for the 29 states and Delhi for children aged 0 to 19 years across three time periods—2005, 2011, and 2017. Currently, GBD is the most reliable source in India, as it collates the data from the national databases of the federal government and extensive multicenter studies in India, including the International Study of Asthma and Allergies in Childhood (ISSAC) and the India Study on Epidemiology of Asthma, Respiratory Symptoms and Chronic Bronchitis (INSEARCH) studies [39,43,45].

#### 2.2.2. Exposures and Other Variables

Area Greenness: Greenness was obtained from Landsat Thematic Mapper Surface Reflectance images (https://earthexplorer.usgs.gov/, accessed on 5 May 2020), which we used to estimate the normalized differential vegetation index (NDVI). The Landsat images constituted the surface reflectance images generated every 16 days at a 30-m pixel spatial resolution and selected images for November. We obtained the next closest image if we missed or could not generate the surface reflectance images for that specific 16-day period. Similarly, the NDVI for each state was estimated across the three time points of 2005, 2011, and 2017. The selected cloud-free (<10 percent clouds) images avoided contamination with the NDVI values. An NDVI value of ‘0′ means no vegetation, and values close to ‘1′ represent the highest greenness [46]. The NDVI captured the density of greenness at a spatial resolution of 30 m and was calculated using the Quantum Geographical Information System (QGIS), Gnu General Public License, Version 2, 1991 Free Software Foundation, Inc. (Boston, MA, USA) tool v.3.12.3 9 [47]. The ratio of visible light (R) to the near-infrared (NIR) light reflected by the vegetative growth comprises the NDVI, that is, the surface reflectance of light during photosynthetic activity [46]. Greenness in a spatial area is defined as an average value of NDVI. The average NDVI values were calculated for the years 2005, 2011, and 2017, representing the annual greenspace exposure for the respective time periods in our study. The NDVI proportions were calculated using the following expression NDVI = (NIR − R)/(NIR + R)”.

Air pollution: The exposure estimates of air pollutants were from open data sources from the statutory organization the Central Pollution Control Board (CPCB) and its counterparts, the State Pollution Control Boards (SPCBs) of India. The annual average concentrations of particulate matter up to diameters of 2.5 µm (PM_2.5_) and 10 µm (PM_10_), nitrogen dioxide (NO_2_), sulfur dioxide (SO_2_), and ozone (O_3_) in µg/m^3^ were from the routine air quality monitoring stations of India for each of the years 2005, 2011, and 2017. We considered a small number of air pollutant data from reports, web pages, and details from the environmental information system (ENVIS) of the central pollution control board and state government. The published literature across 30 states in India contributed to some of the data on the average annual air pollutants, and data with different units of measure, such as parts per billion (ppb), were converted into micrograms per meter cube. Northeast India presents only recent years’ data, as the air quality monitoring stations across all seven states are recent. CPCB had first set the air quality standards in India in 1982, which were further revised by the year 2009, and the standards were higher than the air quality guidelines of WHO [48,49]. The National Air Quality Monitoring Program (NAMP), India, has set the National Ambient Air Quality Standards (NAAQS), with permissible limits for each of the air pollutants to protect public health, vegetation, and property [50].

Weather and other variables: The meteorological data for the states in India were recorded from online points of data collection—www.weatheronline.in, www.wunderground.com, and the Indian Meteorological Department (IMD); accessed on 15 December 2020—spanning 2005, 2011, and 2017. The meteorological parameters utilized were the annual average maximum temperature (degree Celsius), relative humidity (percentage), and average annual rainfall (millimeters). Population distribution in India is measured using the density of the population. Population density (PD) data across all states in 2005, 2011, and 2017 were included from the Census of India website, Refs. [51,52,53], representing the number of persons per square kilometer (km^2^). The Social Progress Index (SPI) is a tool reproduced by the Institute for Competitiveness and Social Progress Imperative that comprises facets of social progress on basic human needs, the foundation of wellbeing, and opportunity, which includes various components [54,55]. The SPI supplements economic success measures by directly measuring social and environmental outcomes. The scores range on a scale of 0–100 for each state. A higher state score indicates a better understanding of the relationship between economic gain and social progress.

### 2.3. Statistical Analysis

We used data visualization methods to illustrate the distribution of asthma prevalence in the India map chart across 29 states and Delhi over the years 2005, 2011, and 2017. The estimated mean NDVI values of the 29 states and 1 union territory (SUTs) projected spatial distributions of average greenness over 2005, 2011, and 2017. Similar graphical methods described the differences in the population rates with PM_2.5_ and O_3_ levels and asthma prevalence. Two-dimensional multiple line graphs were plotted to understand the pattern of association between asthma prevalence and the mean NDVI (*Y*1 and *Y*2-axis) over the periods on the horizontal axis (*X*-axis), created using Stata IC16 software(StataCorp, College Station, TX, USA). Statistical software R, v.3.6.3, was used to plot the multiple variables, using gg-plot for bubble charts and contour plots. The bubble plot is a multi-variable chart similar to a scatterplot and describes the average concentrations of the pollutants and the mean NDVI across the *X*-axis and *Y*-axis.

Spearman’s correlations were estimated to determine the levels of correlation among air pollutants (PM_2.5_, PM_10_, SO_2_, NO_2_, and O_3_), and between air pollutants and meteorological factors (maximum temperature, relative humidity, and average annual rainfall). 

We first separately analyzed the association between the NDVI and the prevalence of asthma in each year (2005, 2011, and 2017), controlling for each air pollutant (PM_2.5_, PM_10_, SO_2_, NO_2_, and O_3_). For each year, the regression model approach that we took was modeling the outcome as a Poisson regression and adjusting it for meteorological parameters, PD, and SPI. We then used a generalized estimating equation (GEE) to estimate the association between the measure of greenness (NDVI) as the primary exposure variable and the prevalence of asthma as the outcome, using a Poisson link and combining the three time points in a single model. Additionally, we also examined the interaction between the NDVI and high air pollutant levels over the three time periods across all states in the GEE model. The air pollutant concentrations higher than the 75th centile were considered high. All statistical tests were 2-sided; effect estimates with a 95% confidence interval (CI) were reported and considered to have strong evidence of association if the p-value was less than 0.05. The statistical data analyses were performed using Stata IC 16 (StataCorp, College Station, TX, USA).

## 3. Results

**Figure 1:**
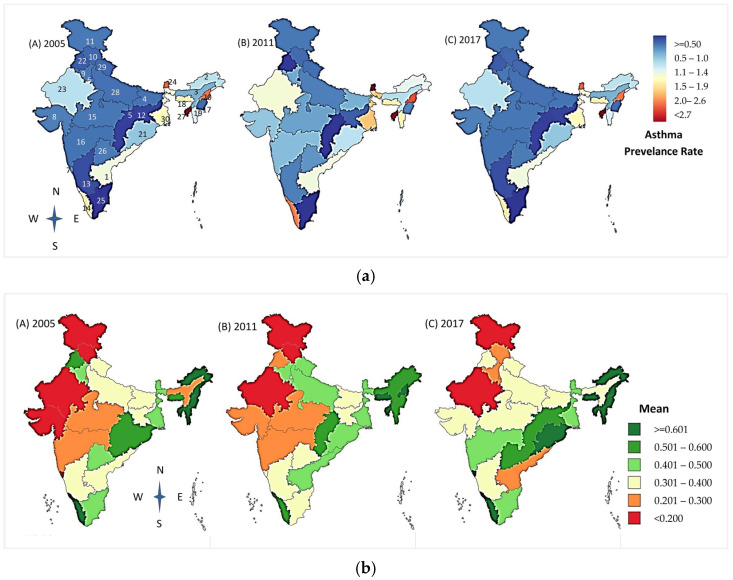
Distribution of (**a**) asthma prevalence rate, (**b**) NDVI across states and union territories in India in 2005, 2011, and 2017.

The overall prevalence (min–max) of childhood and adolescent asthma for 0–19 years was 1275.56 (870.91–2188.52) cases per 100,000 in 2005, 1776.10 (1247.45–2894.67) cases per 100,000 in 2011, and 1419.97 (1128.66–2276.06) cases per 100,000 in 2017, representing an increase of 39.24% and a decrease of 20.05% between the three time periods (Table 1). Asthma in children and adolescents was more prevalent across the northeastern regions of India than in the rest (Figure 1A). The prevalence rates of asthma across all the states in India show a progressive increase from 2005 (1275 cases per 100,000) to 2011 (1776 cases per 100,000), and then present a downward trend until 2017 (1419 cases per 100,000) (Table 1). Though we observed a downward trend, all the states had a higher prevalence of asthma in 2017 compared to 2005 except for Assam (14.49% reduction in prevalence from 2005 to 2017), Goa (8.29%), Kerala (8.90%), Odisha (2.82%), and Meghalaya (2.49%).

**Figure 2:**
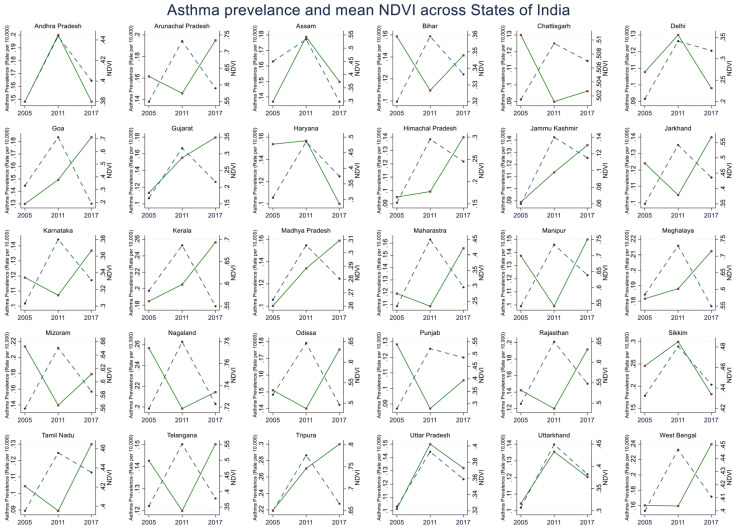
Asthma prevalence rate (per 10,000 population) and mean NDVI across 29 states and 1 union territory (SUTs) of India in 2005, 2011, and 2017.

The overall mean NDVI values in India were 0.43 in 2005, 0.42 in 2011, and 0.41 in 2017 (Figure 1B). The snow-capped and arid desert regions exhibited a low NDVI of around 0.06, and rich forest areas exhibited a high NDVI of 0.80. The greenest states in India with the highest NDVI values in 2005, 2011, and 2017 were in the northeast. The states with the lowest NDVI values included Jammu Kashmir, the northernmost state, which has many snow-capped months in a year; Rajasthan, a predominantly arid and desert area; and Himachal Pradesh, an alpine and subtropical climate. The NDVI values across all the states in India show a small reduction of around 2% over 12 years. There was a concordant change in the prevalence of asthma and the NDVI in the north and northeastern states and in the southern states of Goa, Andhra Pradesh, and Kerala, while divergent changes were present in most other states in India (Figure 2).

**Figure 3:**
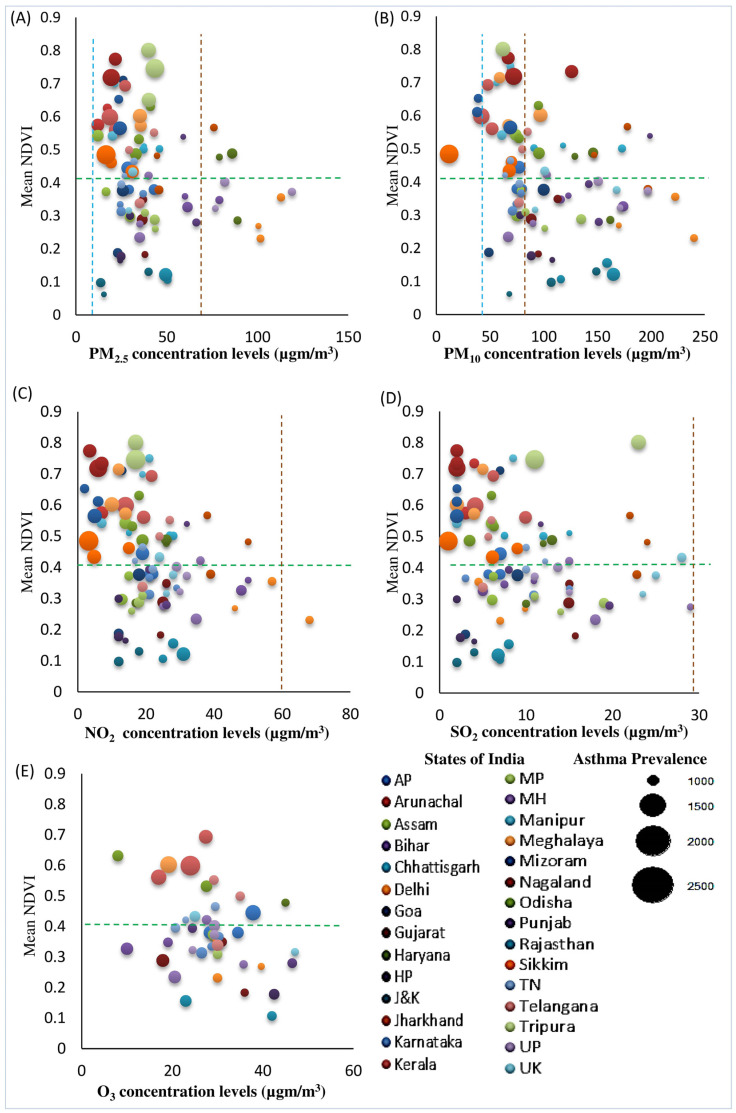
Mean NDVI vs. concentration of air pollutants (**A**) PM_2.5_, (**B**) PM_10_, (**C**) NO_2_, (**D**) SO_2_, and (**E**) O_3_ (in µg/m^3^) across SUTs in the years 2005, 2011, and 2017. Note: The left panel shows the association of the mean NDVI along the Y-axis and the PM_2.5_ concentration levels in the *X*-axis with the asthma prevalence rates across states as bubbles in the three time periods of 2005, 2011, and 2017. The right panel shows the association of the mean NDVI along the *Y*-axis and the PM_10_ concentration levels on the *X*-axis with the asthma prevalence rate across states. Similarly, the middle panel on the left presents the NO_2_ concentration levels, and the middle right presents the SO_2_ concentration levels. The bottom left panel shows the association of the mean NDVI (*Y*-axis) and changes in the concentration levels of O_3_ (*X*-axis) with the prevalence rate of asthma, represented as bubbles in the right panel. Each of the bubbles measures the asthma prevalence rate of each state in the three time periods. Increases in the size of the bubbles represent increases in the asthma prevalence rate and vice-versa. The colors of the bubble represent each state presented on the right side of the panel. Within the graph, the broken blue lines represent the cut-off levels for air pollutant concentration levels as per the World Health Organization (WHO) standards; the brown lines represent the cut-off levels for air pollutants as per the National Ambient Air Quality Standards, India (NAAQS), and the green lines show the mean NDVI values, above which areas show high greenspaces. Note: NDVI, normalized differential vegetation index; PM_2.5_, particulate matter of diameter 2.5 µm or smaller; PM_10_, particulate matter of diameter 10µm or smaller; NO_2_, nitrogen dioxide; SO_2_, sulfur dioxide; O_3_, ozone.

As expected, a strong correlation exists between PM_2.5_ and PM_10_, while there were weak to moderate correlations among the other air pollutants in this study (Supplementary Materials, Table S1). The maximum temperature (temp) levels were positively but weakly correlated with PM_2.5_, PM_10_, and NO_2_. The distributions of the NDVI, asthma rates, and particulate matter (PM_2.5_ and PM_10_) show a moderate negative correlation between the percentage of exposure to particulate matter (PM_2.5_ and PM_10_) and the mean NDVI (Figure 3). Compared to the distribution of particulate matter, many states with exposure to gaseous pollutants (SO_2_ and O_3_) had more variance, with many states showing high exposure (SO_2_ and O_3_) as well as high NDVI values, except for NO_2_ (Figure 3). All 30 states exceeded the ambient PM_2.5_ World Health Organization (WHO) air quality guideline, while 33% of the states exceeded the annual NAAQS (>40 µg/m^3^) in 2005 and 2011, and 50% of states exceeded it in 2017. Kerala (range 42–52 µg/m^3^) is the only state that did not exceed the NAAQS permissible limit (>60 µg/m^3^) for PM_10_ throughout 2005, 2011, and 2017.

The NDVI was associated with increased asthma prevalence rates in all three years (2005, 2011, and 2017) even after adjusting for individual pollutants (Supplementary Materials, Table S2). However, in the models adjusted for O_3_, the NDVI had no association with the asthma prevalence rates in 2011 and 2017.

We also analyzed all three years in one model. The univariate GEE models of high particulate matter, SO_2_, NO_2_, and O_3_ demonstrated a strong negative association with the prevalence of asthma, with only high PM_10_ showing a positive association (Table 2). The evidence of the association of the NDVI and air pollutants with asthma prevalence remained the same when adjusting for the weather parameters, socio-demographic factors such as the SPI, and population density, except for NO_2_ in this study. The NDVI, after adjustment for high levels of PM_2.5_, was positively associated with asthma prevalence (β = 0.144; 95% CI = 0.10, 0.186; *p* < 0.0001). In contrast, there was no association between the NDVI and asthma prevalence after adjustment for PM_10_ at high levels (β = 0.035; 95% CI = −0.006, 0.076; *p* = 0.096). The NDVI in the models, with high levels of both gaseous air pollutants, SO_2_, and NO_2_, was strongly positively associated with asthma prevalence ((β = 0.12; 95% CI = 0.08, 0.16; *p* < 0.0001) (β = 0.09; 95% CI = 0.05, 0.13; *p* < 0.0001)). In the model including high O_3_ levels, the NDVI shows a strong negative association with asthma prevalence (β = 0.19; 95% CI = 0.26, 0.11; *p* < 0.0001). 

**Figure 4:**
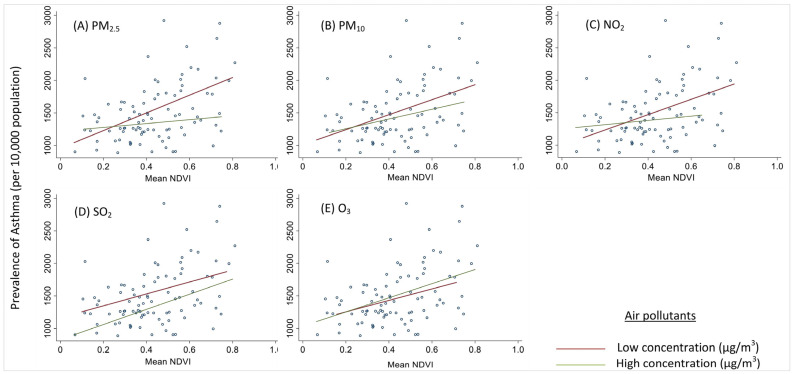
Interaction of the NDVI and air pollutants: (**A**) PM_2.5_, (**B**) PM_10_, (**C**) NO_2_, (**D**) SO_2_, and (**E**) O_3_, influencing the prevalence of asthma. The prevalence rates of asthma in children (0–19 years) in the SUTs are shown on the *Y*-axis with interactions with the mean NDVI (*X*-axis) and low and high concentrations of air pollutants (**A**) PM_2.5_, (**B**) PM_10_, and (**C**) NO_2_ in states in the top panel (left to right). The bottom panel represents the interaction of the mean NDVI (*X*-axis) and high concentrations of air pollutants (**D**) SO_2_ and (**E**) O_3_, represented along the vertical *Y*-axis, with the asthma prevalence rate. Note: NDVI, normalized differential vegetation index; PM_2.5_, particulate matter of diameter 2.5 µm or smaller; PM_10_, particulate matter of diameter 10 µm or smaller; NO_2_, nitrogen dioxide; SO_2_, sulfur dioxide; O_3_, ozone.

The strata-specific effects of low vs. high air pollution on the relationship between the NDVI and asthma prevalence are presented in Figure 4. In all cases except SO_2_, there was an interaction between the air pollutants and the NDVI in the GEE models (Table 3). The largest difference in the slopes can be observed in the PM_2.5_ and NO_2_ plots, where in the states with low concentrations of air pollutants, an increase in the mean NDVI is associated with an increase in asthma prevalence, with a greater rate of change.

Data presented for selected Indian states and union territories of the present study: (1) Andhra Pradesh, (2) Arunachal Pradesh, (3) Assam, (4) Bihar, (5) Chhattisgarh, (6) Delhi, (7) Goa, (8) Gujarat, (9) Haryana, (10) Himachal Pradesh, (11) Jammu and Kashmir, (12) Jharkhand, (13) Karnataka, (14) Kerala, (15) Madhya Pradesh, (16) Maharashtra, (17) Manipur, (18) Meghalaya, (19) Mizoram, (20) Nagaland, (21) Odisha, (22) Punjab, (23) Rajasthan, (24) Sikkim, (25) Tamil Nadu, (26) Telangana, (27) Tripura, (28) Uttar Pradesh, (29) Uttarkhand, and (30) West Bengal. 

In Figure 1A, the rates of asthma prevalence in ages 0 to 19 years are shown on a spectrum of dark blue (0 prevalence) to dark red (highest prevalence) for the years 2005 (left panel), 2011 (middle panel), and 2017 (right panel). In Figure 1B, the normalized differential vegetation index (NDVI) values for 2005 (left), 2011 (middle), and 2017 (bottom), as calculated by the QGIS, are shown on a spectrum of red (least value) to dark green (highest value). These NDVI values relate to the distribution of the average level of greenness across 30 states. Mean NDVI values of 0 to 0.2 are categorized as low, >0.2 to 4.0 as moderate, and >0.4.0 as high levels of greenspaces. The darkest green areas consistently have the highest levels greenspace, while the red areas show the lowest levels of greenspace.

The lines on the graph represent the temporal trends of the series of data (broken blue lines represent the asthma prevalence rates and the green lines show the mean NDVI) over the three time periods of 2005, 2011, and 2017 along the horizontal axis (*X*-axis); the *Y*1-axis on the left is the asthma prevalence rate (blue), and the *Y*2-axis on the right is the mean NDVI (green).

Selected India states and union territories (SUTs) of the present study: (1) Andhra Pradesh, (2) Arunachal Pradesh, (3) Assam, (4) Bihar, (5) Chhattisgarh, (6) Delhi, (7) Goa, (8) Gujarat, (9) Haryana, (10) Himachal Pradesh, (11) Jammu and Kashmir, (12) Jharkhand, (13) Karnataka, (14) Kerala, (15) Madhya Pradesh, (16) Maharashtra, (17) Manipur, (18) Meghalaya, (19) Mizoram, (20) Nagaland, (21) Odisha, (22) Punjab, (23) Rajasthan, (24) Sikkim, (25) Tamil Nadu, (26) Telangana, (27) Tripura, (28) Uttar Pradesh, (29) Uttarkhand, and (30) West Bengal. Note: NDVI, normalized differential vegetation index.

## 4. Discussion

Our findings highlight that asthma prevalence was highest in the northeastern regions, followed by some parts of the eastern coastal plains, while semi-arid areas show low-prevalence estimates. As expected, the northeastern and east coast regions exhibited the highest levels of greenspaces, while the hot arid regions and the snow-capped regions exhibited the lowest levels of greenspaces. The present ecological study is the first state-wide study to show that greenspace is associated with childhood asthma prevalence in India. Our results suggest that greenspaces are positively associated with asthma prevalence in children, though this is not uniform across the geographical regions of India. Although this study enabled us to estimate asthma prevalence trends across the three periods (2005, 2011, and 2017), the established time trend and effect estimates indicate shifts in these trends over those years. Although no studies have assessed greenspace and childhood asthma in India, several studies in other regions have evaluated the effect of greenspace and asthma but reported inconsistent associations. Studies in Spain, New York, and Lithuania have observed that children (9–12 years, 7 years, and 4–6 years) residing close to greenspaces had an increased prevalence of asthma [28,29,32]. In contrast, studies from Cincinnati, Texas, the European region, and Canada have found no such association [56,57,58], and others from Spain, Australia, and Mexico have observed protective effects [27,59,60]. South Asian countries lack extensive studies on greenness and asthma to compare with our findings. 

There are several hypotheses to explain these differences in the observations between greenspaces and their association with asthma. The outcomes, whether adverse (promoting asthma) or protective against asthma, depend on many co-factors. The timing of exposure to greenspaces may be a factor, that is, early-life exposure may have different outcomes than late-life exposure [31,33]. The type of greenspace seems to be relevant; while residences near forest greenspaces are protective, when they are near parks, an increase in the risk of asthma was observed [28]. Several studies have observed that parks and urban spaces may use exotic and non-native trees, which may increase exposure to pollens that have higher allergenicity [29,61]. Additionally, unlike forest greenspaces, there is a higher exposure to pesticides and fertilizers in urban greenspaces [62], increasing the risk of asthma. In some greenspaces, greater exposure to fungal spores increases the risk for asthma [63,64]. Protection from asthma has been observed due to improved air quality [19,22] and improved biodiversity, both at the macrobiota and the microbiota levels [62,63]. Decreased biodiversity has been associated with increased immune system dysfunction [65,66,67]. Higher greenness was associated with decreased air pollution, especially particulate matter, which offered protection in situations of heavy traffic pollution [59] but not in moderate or low traffic pollution. Studies have observed that children residing near a greater density of greenness are engaged in greater physical activity, while children in urban homes with less greenness lead more sedentary lifestyles and are obese, both of which are associated with a greater risk of asthma [68]. Differences in host responses due to various characteristics of the urban built-up environment, especially grey surfaces (which include industries, transport services, and the urban fabric) [69], indoor and outdoor environments, and differences in climate and geography, are all known to influence the complex interaction between greenspaces and asthma. In addition, residents of areas with higher greenness have been observed to have less stress, which impacts the psycho-neuroimmune and hypersensitive reactions to allergens [70,71,72,73]. The differences in the observations may also be due to the variable definitions used to diagnose asthma across studies and variable tools used to quantify greenspaces.

Only a few studies have evaluated the impact of greenspaces and air pollution on asthma, and the evidence is mixed [31,57]. Wide variations have been observed regarding the burden of air pollution, asthma prevalence, and greenspaces over time in different states in diverse countries such as India. When analyzed, a strong positive association was shown between asthma prevalence and greenspaces at high levels of PM_2.5_, PM_10_, NO_2_, and SO_2_, and a strong negative association was shown with O_3_. By examining interactions over time, our study observed the influence of greenspaces and air pollutants (PM_2.5_, PM_10_, SO_2_, NO_2_, and O_3_) on changes in childhood asthma prevalence at different concentration levels of air pollutants. In Australia, greater quantities of greenspace may buffer the impacts of heavy traffic exposure on childhood asthma [59]. Children living in areas with greenspace coverage of more than 40% had a much lower prevalence of asthma than children living in areas with less than 20% greenspace coverage [59]. An eight-year observational study in European (Spain, Germany, and Sweden) birth cohorts showed no associations among asthma, outdoor green environments, and exposure to NO_2_ [58]. The direction of associations among greenspaces, air pollution, and asthma are not always uniform, especially at different time points. In the US (New York), an increase in greenspace was associated with a 29% lower prevalence of asthma in the areas of increased proximity to air pollution sources [27], but a 17% increase in asthma prevalence was observed in areas with high traffic volumes (>1000 vehicles daily) [29]. Similar observations were made by Dadvand et al. in Spain on asthma prevalence, air pollution, and the NDVI at two time points (2012, 2014), and contradictory results have been found [28,31,33]. In another European study (Lithuania), higher greenness levels increased the risk of asthma prevalence when adjusting for PM_2.5_ and NO_2_ [32]. These differences in the direction of association in the same cities (New York, Spain) in two different time periods could be related to differences in the age range of the participants included in the two studies, the diverse analytical sample, the type of study design, the definition of asthma, changes in the quality and quantity of the air pollutants, or the vegetation in the city, similar to other study observations [28,29,32,56,74].

Several potential limitations are important to discuss in our nationwide study. First, the environmental exposure data across the states over all the time points were incomplete due to various reasons, such as, currently, there is a total of 703 air pollutant monitoring stations across all the states compared to 630 stations in the year 2005, while 3000 stations are expected to be established across Indian cities and towns by 2022 [75]. Nevertheless, we managed to retrieve additional data from national and state websites, reports of CPCB, and other studies, leading to uncertainties in the data quality. Still, we used a robust and efficient GEE model to obtain unbiased estimations of the average population. Second, our study presents the findings for the annual average estimates rather than the monthly or daily data, and we might have lost the trends of the short-term effects of environmental exposures. Third, the focus of our study was exploratory, so graphically presenting the differences in the results overcomes this. In addition, using the NDVI tool for estimating greenspaces would have captured some differences in the rural and urban characteristics; however, we would have missed some relative differences in the suburban and urban areas. The NDVI does not account for the vegetation type (denser vs. scarce; natural vs. artificial), and hence, the association may not be detailed; comprehensive assessments can combine different greenspace estimation methods in future research. Studying source-specific roles of each air pollutant [76,77] and their composition was beyond our scope; still, they give a better understanding of the temporal variations along with their distributions across regions.

## 5. Conclusions

In India, greenspace varies over a wide range across states and is associated with increased asthma prevalence, but the association does depend on varying air pollutants in different states. Government initiatives are contributing to green growth, and the smart city initiatives developed in 2017 will continue to further boost the sustained development in urban cities. This is the first study in India, and our study findings contribute to the growing literature on greenspaces and their effects on health under variable environmental factors.

## Figures and Tables

**Table 1:** Summary statistics for the asthma prevalence, environmental factors, and other socio-demographic factors for the years 2005, 2011, and 2017 in states of India.

		2005			2011			2017	
Measures	Mean (SD)	Median (IQR)	Min, Max	Mean (SD)	Median (IQR)	Min, Max	Mean (SD)	Median (IQR)	Min, Max
Asthma prevalence (per 100,000)	1275.559 (±381.06)	1076.84 (1496.51–992.40)	870.90, 2188.52	1776.096 (±480.23)	1609.06 (2038.12–1417.31)	1247.45, 2894.67	1419.96 (±303.84)	1255.00 (1589.02–1219.12)	1419.96, 2276.06
Environmental factors									
NDVI	0.41 (±0.18)	0.41 (0.54–0.27)	0.0621, 0.77	0.41 (±0.15)	0.40 (0.54–0.30)	0.097, 0.746	0.47 (±0.18)	0.42 (0.64–0.34)	0.130, 0.801
Air pollutants									
PM_2.5_ (µgm/m^3^)	37.92 (±20.05)	32.25 (44.9–23.45)	15.4, 119	38.12 (±23.46)	34.05 (44.05–23.17)	12.1, 113	49.41 (±26.52)	40.00 (60.25–31.75)	16.59, 119
PM_10_ (µgm/m^3^)	95.78 (±39.56)	85.00 (117.5–67.25)	39, 199	106.96 (±49.27)	96.34 (149.6–72.5)	12, 222	107.20 (±48.83)	91.00 (145.5–71)	38, 240
SO_2_ (µgm/m^3^)	10.57 (±6.94)	10.00 (15–6)	2, 29	8.92 (±7.07)	6.89 (13.17–2.85)	1, 28	8.73 (±5.91)	7.0 (10.25–5.75)	2, 25
NO_2_(µgm/m^3^)	26.61 (±18.71)	24.00 (30–15)	2, 90	22.25 (±13.97)	19.00 (28.25–12)	3.2, 57	22.14 (±12.43)	21.00 (27.25–14.5)	4.8, 68
O_3_ (µgm/m^3^)	33.51 (±8.96)	40.82	17, 47.1	27.41 (±9.77)	43.56 (31.96–20.66)	10, 46.5	25.47 (±6.12)	46.35 (29.62–22.05)	8, 31
Meteorological variables									
Maximum temperature (degree Celsius)	29.06 (±3.54)	30(31.02–27.63)	16.5, 35	29.18 (±4.23)	29.75 (32–26.78)	16, 35	29.51 (±3.91)	30.25 (32, 27.76)	16, 34.79
Relative humidity (%)	68.75 (±9.13)	69(76.5–61.5)	50, 85	71.13 (±10.33)	70 (80.37–65.5)	50, 90	71.85 (±11.29)	74 (80.5–64.5)	50, 85
Average annual rainfall (mm)	1660.91 (±969.92)	1347.65 (2387.25–1056.75)	520.8, 4692.8	1738.53 (±1099.12)	1359.68 (2378.49–1055.47)	528.13, 5388.8	1601.43 (±903.70)	1297.6 (2624.9–898.37)	299.2, 3443.45
Maximum temperature (degree Celsius)	29.06 (±3.54)	30(31.02–27.63)	16.5, 35	29.18 (±4.23)	29.75 (32–26.78)	16, 35	29.51 (±3.91)	30.25 (32–27.76)	16, 34.79
Socio-economic variables									
Social progress index (SPI) score	48.45 (±5.9)	48.36 (54.72–35.81)	35.81, 59.05	53.85 (±5.9)	53.75 (59.71–49.03)	41.24, 63.04	57.00 (±5.5)	56.38 (62.31–53.43)	44.89, 68.09
Population density (PD) (persons/sqkm)	613.36 (±1665.75)	276.5 (478.5–117.25)	13, 9340	727.16 (±2015.87)	308 (551.25–130)	17, 11297	800.03 (±2217.45)	339 (606.5–143.75)	18, 12427

Note: Data are represented as asthma prevalence, which is cases per 100,000 population; NDVI, normalized differential vegetation index as mean value; PM_2.5_, particulate matter of diameter 2.5 µm or smaller; PM_10_, particulate matter of diameter 10µm or smaller; NO_2_, nitrogen dioxide; SO_2_, sulfur dioxide; O_3_, ozone in µgm/m^3^; maximum temperature in degree Celsius; relative humidity in percentage; average annual rainfall in millimeters; SPI score, social progress index; PD, population density as persons/sqkm; Time 1 is 2005; Time 2 is 2011; Time 3 is 2017; sample size is N; average values as mean (±standard deviation); Min, minimum; Max, maximum; percentile measures at 25th, 50th, 75th, and 90th.

**Table 2:** Generalized estimation equation (GEE) result coefficients (β) (95% CI, lower, upper) of NDVI for association of asthma prevalence and air pollutants at high concentrations.

	Unadjusted	Model 1	Model 2	Model 3	Model 4	Model 5	Model 6
Variables							
NDVI continuous	0.425 (0.390, 0.460) *	0.161 (0.121,0.200) *	0.144 (0.101, 0.186) *	0.035 (−0.006, 0.076)	0.121 (0.082, 0.161) *	0.098 (0.058, 0.138) *	−0.190 (−0.267, −0.113) *
Particulate matter < 2.5 µgm (PM_2.5_), high	−0.137 (−0.153, −0.122) *	−0.094 (−0.111, −0.077) *	−0.051 (−0.070, −0.033) *	..NA	..NA	..NA	..NA
Particulate matter < 10 µgm (PM_10_), high	0.015 (−0.002, −0.028) *	0.037 (0.022, 0.052) *	..NA	0.062 (0.047, 0.078) *	..NA	..NA	..NA
Sulfur dioxide (SO_2_), high	−0.150(−0.163, −0.137) *	−0.102 (−0.116, −0.088) *	..NA	..NA	−0.096 (−0.110, −0.080) *	..NA	..NA
Nitrogen dioxide (NO_2_), high	−0.074 (−0.086, −0.061) *	−0.038 (−0.052, −0.025) *	..NA	..NA	..NA	−0.006 (−0.021, −0.008)	..NA
Ozone (O_3_), high	−0.160 (−0.173, −0.146) *	−0.121 (−0.138, −0.103) *	..NA	..NA	..NA	..NA	−0.118 (−0.135, −0.100) *
Max temperature	0.0008 (−0.0008, 0.0025)	..NA	0.007 (0.006, 0.009) *	0.010 (0.008, 0.012) *	0.006 (0.005, 0.008) *	0.005 (0.003, 0.007) *	0.033 (0.030, 0.037) *
Relative humidity	0.008 (0.008, 0.009) *	..NA	0.006 (0.005, 0.007) *	0.006 (0.005, 0.007) *	0.005 (0.004, 0.006) *	0.005 (0.005, 0.006) *	−3.08 × 10^−4^ (-9.2 × 10^−4^, 9.1× 10^−4^)
Average annual rainfall	1.2 × 10^−5^ (1.2 × 10^−5^, 1.3 × 10^−4^) *	..NA	9.0 x10^−5^ (9.0 × 10^−5^, 1.0 × 10^−5^) *	1.0 × 10^−4^ (1.0 × 10^−4^, 1.1 × 10^−4^) *	9.0 × 10^−5^ (8.0 × 10^−5^, 9.0 × 10^−5^) *	9.0 × 10^−4^ (9.0 × 10^−4^, 1.0 × 10^−4^) *	1.8 × 10^−4^ (1.7 × 10^−4^, 2.0 × 10^−4^) *

Note: Each of the study variables was used in the generalized estimating equation (GEE) with the Poisson link to explore the associations between the rate of asthma prevalence and NDVI (the measure of greenness) as the primary exposure variable. The unadjusted variables are reported separately. In the multivariate analysis, Model 1: Each covariate and weather parameters; further adjusted for maximum temperature, relative humidity, average annual rainfall, social progress index, and population density with each of the air pollutants in Model 2: PM_2.5_; Model 3: PM_10_; Model 4: SO_2_; Model 5:NO_2_; Model 6: O_3_. Air pollutant concentration levels >75th percentile were considered higher, and the controls were variables if less than higher concentration levels. ..NA, not applicable; * indicates significant *p*-values if *p* < 0.05 in univariate, and *p* < 0.001 in multivariate analysis; NDVI, normalized differential vegetation index; PM_2.5_, particulate matter of diameter 2.5 µm or smaller; PM_10_, particulate matter of diameter 10 µm or smaller; NO_2_, nitrogen dioxide.

**Table 3:** GEE interaction analysis—adjusted association between air pollutants and asthma prevalence with NDVI.

			Asthma Prevalence		
Variables	N	Interaction Coefficient (β) (95% CI)	*p*-Int Value	Coefficient (β) (95% CI) of NDVI	*p*-Int Value
PM_2.5_ c.NDVI × PM_2.5_high	82	−0.96 (−1.07, −0.85) *	*p* < 0.001	0.38 (0.33, 0.42) *	*p* < 0.001
PM_10_ c.NDVI × PM_10_high	85	−1.14 (−1.24, −1.04) *	*p* < 0.001	0.261 (0.218, 0.305) *	*p* < 0.001
SO_2_ c.NDVI × SO_2_high	87	−0.03 (−0.12, 0.059)	0.499	0.16 (0.12, 0.20) *	*p* < 0.001
NO_2_ c.NDVI × NO_2_high	87	−1.32 (−1.43, −1.22) *	*p* < 0.001	0.27 (0.23, 0.31) *	*p* < 0.001
O_3_ c.NDVI × O_3_high	45	−0.47 (0.31, 0.62) *	*p* < 0.001	−0.21 (−0.30, −0.12) *	*p* < 0.001

Note: Each of the air pollutants at higher concentration levels with continuous values of NDVI and prevalence of asthma were tested using GEE models via interaction terms with the Poisson link and examined for effects estimates. The Beta coefficient values and 95% confidence intervals are shown. * Indicates significant *p*-interaction values and is reported if *p*-int < 0.1 The model was adjusted for maximum temperature, relative humidity, average annual rainfall, social progress index, and population density.

## Data Availability

The data presented in this study are available upon request from the corresponding author.

## Supplementary Materials

The following supporting information can be downloaded at: https://www.mdpi.com/article/10.3390/ijerph192215273/s1, Table S1: Correlations and *p*-values between the air pollutants and meteorological variables; Table S2: The estimates for asthma prevalence rates and NDVI at each of the air pollutants concentrations for the years 2005, 2011, and 2017.

## References

[1] GINA (2022). Global Strategy for Asthma Management and Prevention.

[2] Soriano J.B., Kendrick P.J., Paulson K.R., Gupta V., Abrams E.M., Adedoyin R.A., Adhikari T.B., Advani S.M., Agrawal A., Ahmadian E. (2020). Prevalence and attributable health burden of chronic respiratory diseases, 1990–2017: A systematic analysis for the Global Burden of Disease Study 2017. Lancet Respir. Med..

[3] Ferrante G., La Grutta S. (2018). The burden of pediatric asthma. Front. Pediatr..

[4] Asher M.I., Rutter C.E., Bissell K., Chiang C.-Y., El Sony A., Ellwood E., Ellwood P., García-Marcos L., Marks G.B., Morales E. (2021). Worldwide trends in the burden of asthma symptoms in school-aged children: Global Asthma Network Phase I cross-sectional study. The Lancet.

[5] Gehring U., Wijga A.H., Brauer M., Fischer P., de Jongste J.C., Kerkhof M., Oldenwening M., Smit H.A., Brunekreef B. (2010). Traffic-related air pollution and the development of asthma and allergies during the first 8 years of life. Am. J. Respir. Crit. Care Med..

[6] Rancière F., Bougas N., Viola M., Momas I. (2017). Early exposure to traffic-related air pollution, respiratory symptoms at 4 years of age, and potential effect modification by parental allergy, stressful family events, and sex: A prospective follow-up study of the PARIS birth cohort. Environ. Health Perspect..

[7] Martinez F.D. (2013). Asthma. Lancet.

[8] Van Tilburg Bernardes E., Arrieta M.-C. (2017). Hygiene hypothesis in asthma development: Is hygiene to blame?. Arch. Med. Res..

[9] Thien F., Beggs P.J., Csutoros D., Darvall J., Hew M., Davies J.M., Bardin P.G., Bannister T., Barnes S., Bellomo R. (2018). The Melbourne epidemic thunderstorm asthma event 2016: An investigation of environmental triggers, effect on health services, and patient risk factors. Lancet Planet Health.

[10] Global Asthma Network (2018). Global Asthma Report 2018.

[11] Singh S., Salvi S., Mangal D.K., Singh M., Awasthi S., Mahesh P.A., Kabra S.K., Mohammed S., Sukumaran T.U., Ghoshal A.G. (2022). Prevalence, time trends and treatment practices of asthma in India: Global Asthma Network study. ERJ Open Res..

[12] García-Marcos L., Asher M.I., Pearce N., Ellwood E., Bissell K., Chiang C.-Y., El Sony A., Ellwood P., Marks G.B., Mortimer K. (2022). The burden of asthma, hay fever and eczema in children in 25 countries: GAN Phase I study. Eur. Respir. J..

[13] Markevych I., Schoierer J., Hartig T., Chudnovsky A., Hystad P., Dzhambov A.M., De Vries S., Triguero-Mas M., Brauer M., Nieuwenhuijsen M.J. (2017). Exploring pathways linking greenspace to health: Theoretical and methodological guidance. Environ. Res..

[14] Lambert K., Bowatte G., Tham R., Lodge C., Prendergast L., Heinrich J., Abramson M.J., Dharmage S., Erbas B. (2017). Residential greenness and allergic respiratory diseases in children and adolescents–a systematic review and meta-analysis. Environ. Res..

[15] Bowatte G., Lodge C.J., Knibbs L.D., Erbas B., Perret J.L., Jalaludin B., Morgan G.G., Bui D.S., Giles G.G., Hamilton G.S. (2018). Traffic related air pollution and development and persistence of asthma and low lung function. Environ. Int..

[16] Evans K.A., Halterman J.S., Hopke P.K., Fagnano M., Rich D.Q. (2014). Increased ultrafine particles and carbon monoxide concentrations are associated with asthma exacerbation among urban children. Environ. Res..

[17] Anenberg S.C., Henze D.K., Tinney V., Kinney P.L., Raich W., Fann N., Malley C.S., Roman H., Lamsal L., Duncan B. (2018). Estimates of the Global Burden of Ambient PM2.5, Ozone, and NO2 on Asthma Incidence and Emergency Room Visits. Environ. Health Perspect..

[18] Pawankar R., Wang J.-Y., Wang I.-J., Thien F., Chang Y.-S., Latiff A.H.A., Fujisawa T., Zhang L., Thong B.Y.-H., Chatchatee P. (2020). Asia Pacific Association of Allergy Asthma and Clinical Immunology White Paper 2020 on climate change, air pollution, and biodiversity in Asia-Pacific and impact on allergic diseases. Asia Pac. Allergy.

[19] Lelieveld J., Evans J.S., Fnais M., Giannadaki D., Pozzer A. (2015). The contribution of outdoor air pollution sources to premature mortality on a global scale. Nature.

[20] WHO Air Pollution. https://www.who.int/health-topics/air-pollution?.

[21] Cohen A.J., Brauer M., Burnett R., Anderson H.R., Frostad J., Estep K., Balakrishnan K., Brunekreef B., Dandona L., Dandona R. (2017). Estimates and 25-year trends of the global burden of disease attributable to ambient air pollution: An analysis of data from the Global Burden of Diseases Study 2015. Lancet.

[22] Erbas B., Kelly A.-M., Physick B., Code C., Edwards M. (2005). Air pollution and childhood asthma emergency hospital admissions: Estimating intra-city regional variations. Int. J. Environ. Health Res..

[23] O’Connor G.T., Neas L., Vaughn B., Kattan M., Mitchell H., Crain E.F., Evans Iii R., Gruchalla R., Morgan W., Stout J. (2008). Acute respiratory health effects of air pollution on children with asthma in US inner cities. J. Allergy Clin. Immunol..

[24] Guarnieri M., Balmes J.R. (2014). Outdoor air pollution and asthma. Lancet.

[25] Khilnani C.G., Tiwari C.P. (2018). Air pollution in India and related adverse respiratory health effects: Past, present, and future directions. Curr. Opin. Pulm. Med..

[26] WHO Concentrations of Fine Particulate Matter (PM2.5). https://www.who.int/data/gho/data/indicators/indicator-details/GHO/concentrations-of-fine-particulate-matter-(pm2-5).

[27] Lovasi G.S., Quinn J.W., Neckerman K.M., Perzanowski M.S., Rundle A. (2008). Children living in areas with more street trees have lower prevalence of asthma. J. Epidemiol. Community Health.

[28] Dadvand P., Villanueva C.M., Font-Ribera L., Martinez D., Basagaña X., Belmonte J., Vrijheid M., Gražulevičienė R., Kogevinas M., Nieuwenhuijsen M.J. (2014). Risks and benefits of green spaces for children: A cross-sectional study of associations with sedentary behavior, obesity, asthma, and allergy. Environ. Health Perspect..

[29] Lovasi G.S., O’Neil-Dunne J.P.M., Lu J.W.T., Sheehan D., Perzanowski M.S., MacFaden S.W., King K.L., Matte T., Miller R.L., Hoepner L.A. (2013). Urban tree canopy and asthma, wheeze, rhinitis, and allergic sensitization to tree pollen in a New York City birth cohort. Environ. Health Perspect..

[30] Ferrante G., Asta F., Cilluffo G., De Sario M., Michelozzi P., La Grutta S. (2020). The effect of residential urban greenness on allergic respiratory diseases in youth: A narrative review. World Allergy Organ. J..

[31] Dadvand P., Sunyer J., Basagana X., Ballester F., Lertxundi A., Fernandez-Somoano A., Estarlich M., Garcia-Esteban R., Mendez M.A., Nieuwenhuijsen M.J. (2012). Surrounding greenness and pregnancy outcomes in four Spanish birth cohorts. Environ. Health Perspect..

[32] Andrusaityte S., Grazuleviciene R., Kudzyte J., Bernotiene A., Dedele A., Nieuwenhuijsen M.J. (2016). Associations between neighbourhood greenness and asthma in preschool children in Kaunas, Lithuania: A case–control study. BMJ open.

[33] Dadvand P., de Nazelle A., Triguero-Mas M., Schembari A., Cirach M., Amoly E., Figueras F., Basagaña X., Ostro B., Nieuwenhuijsen M. (2012). Surrounding greenness and exposure to air pollution during pregnancy: An analysis of personal monitoring data. Environ. Health Perspect..

[34] United Nations (2017). World Population Prospects: The 2017 Revision, Key Findings and Advance Tables.

[35] GBD Chronic Respiratory Disease Collaborators (2017). Global, regional, and national deaths, prevalence, disability-adjusted life years, and years lived with disability for chronic obstructive pulmonary disease and asthma, 1990–2015: A systematic analysis for the Global Burden of Disease Study 2015. Lancet. Respir. Med..

[36] WHO Ambient Air Pollution Data. https://www.who.int/data/gho/data/themes/air-pollution/ambient-air-pollution.

[37] IQAir (2020). World Air Quality Report: Region & City PM2.5 Ranking.

[38] Chowdhury S., Dey S. (2016). Cause-specific premature death from ambient PM2. 5 exposure in India: Estimate adjusted for baseline mortality. Environ. Int..

[39] Salvi S., Kumar G.A., Dhaliwal R.S., Paulson K., Agrawal A., Koul P.A., Mahesh P.A., Nair S., Singh V., Aggarwal A.N. (2018). The burden of chronic respiratory diseases and their heterogeneity across the states of India: The Global Burden of Disease Study 1990–2016. Lancet Glob. Health.

[40] Chokshi M., Patil B., Khanna R., Neogi S.B., Sharma J., Paul V.K., Zodpey S. (2016). Health systems in India. J. Perinatol..

[41] Institute for Health Metrics and Evaluation GBD Compare. http://vizhub.healthdata.org/gbd-compare.

[42] Indian Council of Medical Research Public Health Foundation of India Institute of Health Metrics and Evaluation (2019). GBD India Compare Data Visualization.

[43] Lai C.K., Beasley R., Crane J., Foliaki S., Shah J., Weiland S., Group I.P.T.S. (2009). Global variation in the prevalence and severity of asthma symptoms: Phase three of the International Study of Asthma and Allergies in Childhood (ISAAC). Thorax.

[44] Sinha S., Singh J., Kumar Jindal S., Birbian N. (2015). Association of IL13R alpha 1+ 1398A/G polymorphism in a North Indian population with asthma: A case-control study. Allergy Rhinol. Provid..

[45] Jindal S.K., Aggarwal A.N., Gupta D., Agarwal R., Kumar R., Kaur T., Chaudhry K., Shah B. (2012). Indian Study on Epidemiology of Asthma, Respiratory Symptoms and Chronic Bronchitis in adults (INSEARCH). Int. J. Tuberc. Lung Dis..

[46] Weier J., Herring D. Measuring Vegetation (NDVI & EVI). http://earthobservatory.nasa.gov/Features/MeasuringVegetation.

[47] QGIS Development Team Open Source Geospatial Foundation Project Quantum Geographic Information System. https://www.qgis.org.

[48] Ministry of Environment Forest and Climate Change (2009). Revised National Ambient Air Quality Standards (NAAQS) 2009 Notified. http://moef.gov.in/wp-content/uploads/2019/10/Press-Note-on-RNAAQS_1.pdf.

[49] Central Pollution Control Board India National Ambient Air Quality Standards Central Pollution Control Board Notification in the Gazette of India, Extraordinary. http://www.cpcb.nic.in/upload/Latest/Latest_48_FINAL_AIR_STANDARD.pdf.

[50] Central Pollution Control Board National Ambient Air Quality Status and Trends 2019. 2019, 156..

[51] Government of India Census of India 2011. http://dataforall.org/dashboard/censusinfoindia_pca/.

[52] Government of India (2011). Census of India 2011; 00-001-2011-Cen-Book (E).

[53] Ministry of Statistics and Program Implementation (2018). Children in India 2018—A Statistical Appraisal.

[54] Kapoor A., Kapoor M., Krylova P. (2017). Social Progress Index: States of India 2005–2016, Methodology Report.

[55] Kapoor A., Kapoor M., Krylova P. (2017). Social Progress Index: States of India 2005–2016, Eleven Years of Progress Report Findings.

[56] Pilat M.A., McFarland A., Snelgrove A., Collins K., Waliczek T.M., Zajicek J. (2012). The effect of tree cover and vegetation on incidence of childhood asthma in metropolitan statistical areas of Texas. HortTechnology.

[57] Sbihi H., Koehoorn M., Tamburic L., Brauer M. (2017). Asthma trajectories in a population-based birth cohort. Impacts of air pollution and greenness. Am. J. Respir. Crit. Care Med..

[58] Tischer C., Dadvand P., Basagana X., Fuertes E., Bergström A., Gruzieva O., Melen E., Berdel D., Heinrich J., Koletzko S. (2018). Urban upbringing and childhood respiratory and allergic conditions: A multi-country holistic study. Environ. Res..

[59] Feng X., Astell-Burt T. (2017). Is neighborhood green space protective against associations between child asthma, neighborhood traffic volume and perceived lack of area safety? Multilevel analysis of 4447 Australian children. Int. J. Environ. Res. Public Health.

[60] Eldeirawi K., Kunzweiler C., Zenk S., Finn P., Nyenhuis S., Rosenberg N., Persky V. (2019). Associations of urban greenness with asthma and respiratory symptoms in Mexican American children. Ann. Allergy Asthma Immunol..

[61] DellaValle C.T., Triche E.W., Leaderer B.P., Bell M.L. (2012). Effects of ambient pollen concentrations on frequency and severity of asthma symptoms among asthmatic children. Epidemiology.

[62] Corsini E., Sokooti M., Galli C.L., Moretto A., Colosio C. (2013). Pesticide induced immunotoxicity in humans: A comprehensive review of the existing evidence. Toxicology.

[63] Bartra J., Belmonte J., Torres-Rodriguez J.M., Cistero-Bahima A. (2009). Sensitization to Alternaria in patients with respiratory allergy. Front. Biosci..

[64] De Linares C., Belmonte J., Canela M., de la Guardia C.D., Alba-Sanchez F., Sabariego S., Alonso-Pérez S. (2010). Dispersal patterns of Alternaria conidia in Spain. Agric. For. Meteorol..

[65] Rook G.A. (2013). Regulation of the immune system by biodiversity from the natural environment: An ecosystem service essential to health. Proc. Natl. Acad. Sci. USA.

[66] Hanski I., von Hertzen L., Fyhrquist N., Koskinen K., Torppa K., Laatikainen T., Karisola P., Auvinen P., Paulin L., Mäkelä M.J. (2012). Environmental biodiversity, human microbiota, and allergy are interrelated. Proc. Natl. Acad. Sci. USA.

[67] Mhuireach G., Johnson B.R., Altrichter A.E., Ladau J., Meadow J.F., Pollard K.S., Green J.L. (2016). Urban greenness influences airborne bacterial community composition. Sci. Total Environ..

[68] Baiardini I., Sicuro F., Balbi F., Canonica G.W., Braido F. (2015). Psychological aspects in asthma: Do psychological factors affect asthma management?. Asthma Res. Pract..

[69] Tischer C., Gascon M., Fernández-Somoano A., Tardón A., Materola A.L., Ibarluzea J., Ferrero A., Estarlich M., Cirach M., Vrijheid M. (2017). Urban green and grey space in relation to respiratory health in children. Eur. Respir. J..

[70] Astell-Burt T., Feng X., Kolt G.S. (2013). Mental health benefits of neighbourhood green space are stronger among physically active adults in middle-to-older age: Evidence from 260,061 Australians. Prev. Med..

[71] Chen E., Schreier H.M.C., Strunk R.C., Brauer M. (2008). Chronic traffic-related air pollution and stress interact to predict biologic and clinical outcomes in asthma. Environ. Health Perspect..

[72] Islam M.N., Rahman K.-S., Bahar M.M., Habib M.A., Ando K., Hattori N. (2012). Pollution attenuation by roadside greenbelt in and around urban areas. Urban For. Urban Green..

[73] Shankardass K., McConnell R., Jerrett M., Milam J., Richardson J., Berhane K. (2009). Parental stress increases the effect of traffic-related air pollution on childhood asthma incidence. Proc. Natl. Acad. Sci. USA.

[74] Sbihi H., Tamburic L., Koehoorn M., Brauer M. (2015). Greenness and Incident Childhood Asthma: A 10-Year Follow-up in a Population-based Birth Cohort. Am. J. Respir. Crit. Care Med..

[75] Indian Institute of Management (2010). Evaluation of Central Pollution Control Board (CPCB).

[76] Srivastava A., Gupta S., Jain V.K. (2008). Source Apportionment of Total Suspended Particulate Matter in Coarse and Fine Size Ranges Over Delhi. Aerosol Air Qual. Res..

[77] Saxena M., Sharma A., Sen A., Saxena P., Saraswati, Mandal T.K., Sharma S.K., Sharma C. (2017). Water soluble inorganic species of PM10 and PM2.5 at an urban site of Delhi, India: Seasonal variability and sources. Atmos. Res..

