# Supplementary material for: Time Trends of Greenspaces, Air Pollution, and Asthma Prevalence among Children and Adolescents in India
Source: Int J Environ Res Public Health. 2022 Nov 18;19(22):15273. doi: 10.3390/ijerph192215273 (PMC9690186; doi:10.3390/ijerph192215273)
Supplement: Supplementary file 1 [file ijerph-19-15273-s001.zip › ijerph-1937473-supplementary.pdf]

Table S1. Correlations and p−values between the air pollutants and meteorological variables

	$PM_{2.5}$	PM_{10}	SO_2	NO_2	O_3	Maximum temperature	Relative humidity	Average Annual Rainfall
$PM_{2.5}$	1							
PM_{10}	**0.809**	1						
	0.000							
SO_2	**0.230**	**0.330**	1					
	0.041	0.002						
NO_2	**0.494**	**0.535**	**0.332**	1				
	0.000	0.000	0.001					
O_3	0.068	0.049	0.148	−0.067	1			
	0.683	0.747	0.148	0.662				
Maximum temperature	0.119	−0.069	0.172	0.206	−0.091	1		
	0.284	0.530	0.111	0.055	0.548			
Relative humidity	−0.192	**−0.236**	**−0.284**	**−0.246**	**−0.314**	**−0.298**	1	
	0.0825	0.0294	0.0077	0.0217	0.0353	0.0043		
Average Annual Rainfall	**−0.4049**	**−0.4787**	**−0.3280**	**−0.3079**	**−0.3580**	0.0063	**0.4687**	1
	0.0002	0.0000	0.0019	0.0037	0.0157	0.9530	0.0000	

Note: Data describes the Correlation matrix for the air pollutants and the meteorological factors. The corresponding p values are reported below each correlation co−efficient values. Significant p values that is $p<0.05$ are made bold. The variables are continuous represent $PM_{2.5}$, particulate matter of diameter 2.5μm or less; PM_{10}, particulate matter of diameter 10μm or less; NO_2, nitrogen dioxide; SO_2, sulfur dioxide; and, O_3, ozone measured as $\mu gm/m^3$; maximum temperature in degree Celsius; Relative Humidity in percentage; Average Annual Rainfall in millimeter.

Table S2. The estimates for asthma prevalence rates and NDVI with each of the air pollutants concentrations for the year 2005, 2011, and 2017.

		Asthma Prevalence Rates (95% CI)		
		2005	2011	2017
**NDVI continuous**				
	Unadjusted	1.44 (1.35, 1.54)	1.95 (1.83, 2.09)	1.05 (0.97, 1.15)
	Particulate matter <2.5µgm ($PM_{2.5}$)	1.45 (1.36, 1.54)	1.92 (1.80, 2.05)	1.17 (1.05, 1.30)
	Particulate matter <10µgm (PM_{10})	1.39 (1.30, 1.49)	1.24 (1.15, 1.34)	1.12 (1.02, 1.22)
	Sulfur dioxide (SO_2)	1.41 (1.31, 1.52)	1.91 (1.79, 2.04)	1.06 (0.97, 1.15)
	Nitrogen dioxide(NO_2)	1.50 (1.39, 1.61)	1.95 (1.81, 2.10)	1.11 (1.01, 1.20)
	Ozone (O_3)	2.01 (1.74, 2.33)	1.24 (0.99, 1.55)	0.80 (0.67, 0.94)

Note: The model estimated the association between continuous NDVI and asthma prevalence rate for 2005, 2011 and 2017, adjusting for each air pollutant $PM_{2.5}$, PM_{10}, NO_2, SO_2 and O_3 concentration. The asthma prevalence rates and 95% confidence intervals (95% CI) are shown. NDVI: normalized difference vegetation index. All models are adjusted for maximum temperature, relative humidity, average annual rainfall, social progress index, and population.
